# Cariprazine augmentation of clozapine in schizophrenia—a retrospective chart review

**DOI:** 10.3389/fphar.2023.1321112

**Published:** 2024-01-04

**Authors:** Marcin Siwek, Adrian Andrzej Chrobak, Aleksandra Gorostowicz, Patrycja Król, Dominika Dudek

**Affiliations:** ^1^ Department of Affective Disorders, Jagiellonian University Medical College, Kraków, Poland; ^2^ Department of Adult Psychiatry, Jagiellonian University Medical College, Kraków, Poland

**Keywords:** clozapine resistance, negative symptoms, positive symptoms, psychosis, pharmacotherapy, antipsychotic drugs

## Abstract

The aim of our study was to evaluate the efficacy of cariprazine augmentation of clozapine in treatment-resistant schizophrenia in a retrospective chart review. Among 916 medical records of schizophrenia patients, we identified 12 individuals treated with a combination of those drugs for a duration of 3–60 weeks [median 32 (10–40)]. Clinical Global Impression–Improvement (CGI-I) scores were used to measure the treatment response between the introduction of cariprazine augmentation of clozapine and the last point of observation. The majority of the patients presented treatment response (9/12 patients, 75%) after 4–16 weeks of therapy [median 6 (4–12)]. Treatment was associated with the decrease in positive, negative, affective, and anxiety symptom severity, as well as improvement of patient global functioning. One patient discontinued the treatment due to side effects (akathisia), and two patients halted the therapy due to the exacerbation of psychotic symptoms. Our study presents a thorough clinical description of the largest number of treatment-resistant schizophrenia patients medicated using cariprazine augmentation of clozapine in a “real-world” setting. Our results suggest that the use of this combination may lead to the improvement in a broad range of symptoms of patients with this condition.

## 1 Introduction

Schizophrenia is a chronic and devastating mental illness characterized by the occurrence of negative, positive, and cognitive symptoms (Suttajit et al., 2015). Despite the observable progress in the field of psychopharmacotherapy, approximately 30%–50% of patients still present treatment resistance, which is defined as the persistence of psychotic symptoms after at least two subsequential antipsychotic drug monotherapies with an adequate dose, duration, and patient compliance (Nucifora et al., 2019; Correll and Howes, 2021). The only medicine that received FDA recommendation in the case of treatment-resistant schizophrenia is clozapine, which has been shown to be the most effective antipsychotic drug for reducing positive symptoms, suicidal risk, and the frequency and duration of hospitalizations (Kane et al., 1988; Chakos et al., 2001; Siskind et al., 2016; 2019; Wimberley et al., 2017; Cho et al., 2019). Even though clozapine has been shown to be more effective than other neuroleptics, as many as 40%–70% of patients do not achieve a satisfactory response to the treatment with the use of this drug (Rajkumar et al., 2013; McCutcheon et al., 2015; Sagud, 2015; Chakrabarti, 2021). A common strategy to overcome clozapine resistance is augmentation using a second antipsychotic drug. However, data supporting this method come from low-quality studies, e.g., open-label trials or case studies (Wagner et al., 2019). A recent meta-analysis of randomized controlled trials evaluating treatment strategies for clozapine-resistant schizophrenia showed that none of the analyzed neuroleptics (aripiprazole, amisulpride, sulpiride, risperidone, sertindole, pimozide, and quetiapine) differed from placebo (Yeh et al., 2023). The use of more modern antipsychotics such as lurasidone and cariprazine as a method of clozapine augmentation is limited (De Berardis et al., 2021; Oloyede et al., 2022; Pappa et al., 2022; Weise et al., 2022; Siwek et al., 2023).

Cariprazine is a novel antipsychotic drug characterized by D_2_/D_3_ partial agonism with almost 10-fold higher affinity to D_3_ than D_2_ receptors, as well as partial agonistic activity at serotonin 5-HT_1a_ receptors (Misiak et al., 2018). Table 1 summarizes the affinities of cariprazine and its clinical effects compared to other partial D_2_ agonists (Siwek et al., 2023). Due to its unique pharmacokinetic features, this drug has been referred to as an “oral depot,” as the half-time of cariprazine ranges between 2 and 4 days, and in the case of its metabolite didesmethyl cariprazine, it may be up to even 2–3 weeks (Misiak et al., 2018; Oloyede et al., 2022). The efficacy of the monotherapy using this drug in schizophrenia has been proved in several short-term randomized controlled trials and a double-blinded extension study (Misiak et al., 2018). Recent studies suggest that due to the abovementioned unique pharmacokinetic and pharmacodynamic properties, cariprazine may also be beneficial in combination with other antipsychotics (Hjorth 2021; Boydstun et al., 2023). Its noticeable effectiveness has been shown in the area of negative symptoms. In a 26-week placebo-controlled double-blinded study, performed on a group of 461 schizophrenia patients, cariprazine monotherapy has shown high efficacy in the treatment of persistent and predominant negative symptoms compared to risperidone (Németh et al., 2017). Taking into account the frequent occurrence of these symptoms in treatment-resistant schizophrenia and the small effect of clozapine on their improvement, the use of cariprazine for its augmentation may be beneficial (Krause et al., 2018; Correll and Schooler, 2020). While there are many studies evaluating the efficacy of the augmentation of clozapine with the use of other antipsychotics in treatment-resistant schizophrenia (Yeh et al., 2023), data concerning the use of cariprazine for this purpose are limited to case reports and one observational study (De Berardis et al., 2021; Oloyede et al., 2022; Pappa et al., 2022). Authors have suggested that cariprazine may be uniquely effective in combinations with other antipsychotic drugs. Pappa et al. (2022) performed a pilot prospective study evaluating the efficacy and safety of this combination in a group of patients with sub-optimal treatment response to clozapine. The study was performed on a heterogeneous group of 10 patients diagnosed with schizophrenia, schizoaffective disorder, and personality disorder, in which augmentation with cariprazine was introduced due to the presence of negative symptoms, treatment resistance, and/or tolerability issues with clozapine or previous augmentation options. The authors observed a reduction in both positive and negative symptom scores after 3 months of therapy, indicating good tolerance to the treatment. Due to those results indicating a beneficial effect of cariprazine augmentation of clozapine, the purpose of our study was to evaluate the effectiveness of this combination in treatment-resistant schizophrenia by performing a retrospective chart review.

**TABLE 1:** Comparison of affinities of cariprazine and other partial agonists of D_2_ for specific receptors. Additionally, the clinical effects of agonism and antagonism of those receptors are summarized based on the study by Siwek et al. (2023) with some modifications.

	Effects of receptor agonism/partial agonists	Effects of receptor antagonism and reversed agonism	Brexpiprazole	Aripiprazole	Cariprazine
D_2_	Partial agonism: ↓ EPS	Antipsychotic and antimanic effects	0.30#	0.34#	0.49#
	↓ hPRL	↓ Agitation			
	↓ Risk of seizures	↓ Aggressive behaviors			
		↑ EPS			
		↑ PRL			
		↑ Cognitive impairments			
		↑ Negative symptoms			
		↑ Weight			
		↑ Risk of seizures			
D_3_	↓ Negative symptoms	Possible antipsychotic, antidepressant, and procognitive effects	1.1#	0.8#	0.085#
5HT_1a_	Antidepressant, anxiolytic, and procognitive effects	?	0.12#	1.7#	2.6#
	↓ EPS				
	↑ Sexual impairments associated with serotonin reuptake inhibition				
5HT_2a_	Propsychotic and possible antipsychotic effects	Antipsychotic, anxiolytic, and non-rapid eye movement sleep-promoting effects	0.47*	3.4*	18.8*
	↑ Impulsive behaviors	↓ PRL			
	↑ Insomnia	↓ EPS and impulsive behaviors. Potentialization of the serotonin reuptake inhibition effects			
	↑ Neuroplasticity ↑ Risk of inducing serotonin syndrome	↑ Sedation and insulin resistance			
5HT_2c_	Anorectic effect. ↓ of impulsive behaviors	Procognitive and antidepressant effects	34*	15#	134*
		↑ Weight gain			
5HT_7_	?	Antidepressant, antipsychotic, and procognitive effects	3.7*	29#	111*
		↓ Negative symptoms			
		↓ Duration of rapid eye movement sleep			
α_1_	Hypertension	Hypotonia (including orthostatic hypotonia), tachycardia, nasal swelling, ejaculation impairments, and sedation	3.8*	57*	6.88*
		↑ Weight			
		↑ Risk of metabolic syndrome, priapism, urinary incontinence, and proconvulsive effects			
α_2_	Procognitive and analgesic effects	Antidepressant and possible procognitive effects	0.59*	37.9*	?
	↑ Sedation hypotonia	↑ Nightmares			
	↑↓ Risk of seizures dependent on the drug dose				
H_1_	?	↑ Sedation	19*	61*	23.2*
		↑ Weight			
		Possible ↑ risk of seizures			
M	Procognitive, proconvulsive, and possible antipsychotic effects	↓ EPS	>1,000	>1,000	>1,000
	↑ EPS. Possible sialorrhea	↓ Cognitive functions			
		↑ Agitation			
		↑ Possible psychotic symptoms			
		Peripheral anticholinergic symptoms			

Antagonism, *; partial agonism, #; unknown, ?. The values presented in the table are values of the dissociation constant Ki (nM); the lower the value, the higher the affinity for the receptor. EPS, extrapyramidal symptoms; PRL, prolactin.

## 2 Materials and methods

**FIGURE 1:**
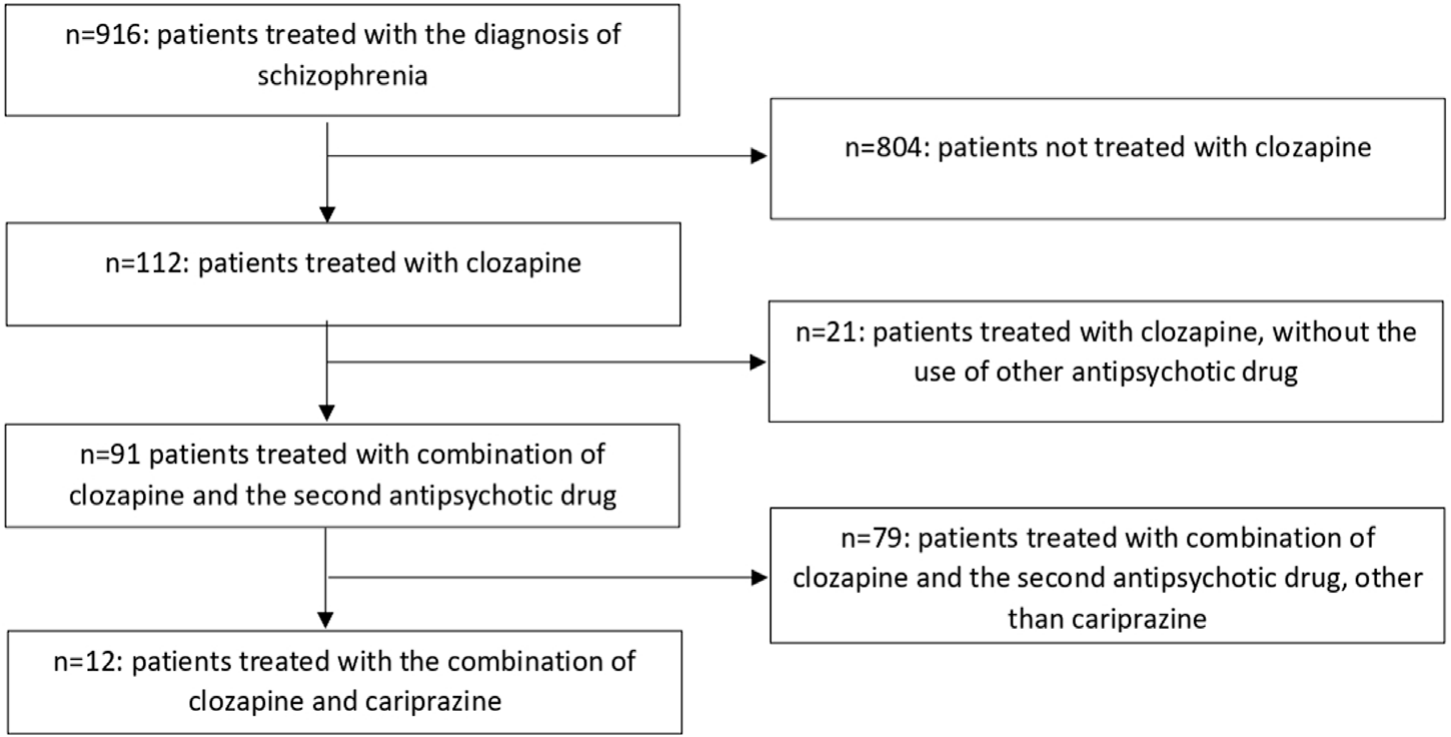
Flowchart of the retrospective chart review.

In order to evaluate the efficacy of cariprazine augmentation of clozapine in treatment-resistant schizophrenia, we performed a retrospective chart review according to the methodology of our previous studies (Siwek et al., 2021; 2023). All the authors analyzed the data. The dataset consisted of medical records (paper and electronic) of all schizophrenia patients (diagnosed in accordance with the ICD-10 criteria) treated in the Department of Adult Psychiatry of University Hospital in Krakow between 2018 and 2022. The data were included in the analysis if they met the following inclusion criteria: diagnosis of schizophrenia, age above 18 years, and treatment with a combination of clozapine and cariprazine. The exclusion criteria were as follows: no documented follow-up observations and missing data relevant to the analysis. Figure 1 shows a flowchart of the retrospective chart review.

Before carrying out data abstraction, the researchers were trained in order to establish a common procedure for the data analysis. The authors organized regular meetings in order to discuss potential doubts occurring during the abstraction process. In the cases of incomplete or missing data, the researchers contacted the psychiatrist who was taking care of the particular patient. Data-collecting forms were based on the methodology of our previous study and are provided with this article.

The authors screened medical records of 916 patients diagnosed with schizophrenia. Among these, 112 were treated using clozapine, and 91 of those patients were treated using an additional neuroleptic. In this group, 12 patients were treated using a clozapine and cariprazine combination. Similar to our previous study, we achieved high inter-rater reliability, as there were only several features on which authors disagreed during data abstraction (Cohen’s kappa >0.8 for all variables). The disagreements were resolved by the discussion. The intra-rater reliability was assessed on a sample of 10 patients, for which Cohen’s kappa was also very high with values >0.8.

Data were extracted from medical records through the use of chart files in the form of an electronic table with the following variables: age, sex, duration of illness, the number of previous ineffective pharmacotherapy trials prior to the use of clozapine and cariprazine combination, dose of clozapine that was combined with cariprazine, antipsychotics used in combination with clozapine prior to the switch to cariprazine, somatic comorbidities, presence of substance abuse according to the ICD-10 classifications, other psychotropic medications used at the time of cariprazine augmentation of clozapine, non-psychiatric medications with their daily doses, initial and final doses of cariprazine, duration of the combined treatment (clozapine and cariprazine) in weeks, the number of weeks until the observable therapeutic effect was achieved, and effects of the addition of cariprazine. Moreover, we evaluated data for the occurrence of the following psychopathological symptoms and somatic conditions presented by the patients before initiation of cariprazine augmentation of clozapine: residual positive symptoms, exacerbation of positive symptoms, negative symptoms, depressive symptoms, anxiety, suicidal thoughts, cognitive dysfunctions, hyperprolactinemia, increased appetite and body weight/obesity, and disorders of glucose metabolism.

We calculated Clinical Global Impression-Improvement (CGI-I) scores with the aim to evaluate the treatment response between the first and last points of observation. The patients were classified as responding to cariprazine augmentation of clozapine if they achieved 1 or 2 points on the CGI-I scale (“Very much improved” or “Much improved”) at any point of the follow-up. In the case of every patient fulfilling the abovementioned criteria, we presented the number of weeks of treatment after which therapeutic effect was observable.

## 3 Results

Data extracted from the medical records of 12 patients treated with the combination of cariprazine and clozapine are given in Table 2 and summarized in Table 3. The patients’ median age was 38 (range 23–59). There were seven men and five women of Caucasian ethnicity. The median duration of illness was 16.5 (range 5–30). All of the patients fulfilled the criteria of treatment-resistant schizophrenia, defined as the persistence of psychotic symptoms after at least two subsequential antipsychotic drug monotherapies with an adequate dose, duration, and patient compliance (Nucifora et al., 2019; Correll and Howes, 2021). The minimal number of previous ineffective psychopharmacotherapy trials prior to the use of cariprazine augmentation of clozapine was four. Four patients underwent more than 10 unsuccessful trials. Table 4 shows the psychopathological symptoms and somatic conditions presented before the initiation of the evaluated therapy.

**TABLE 2:** Detailed description of treatment-resistant schizophrenia patients treated using cariprazine augmentation of clozapine.

Case number	1	2	3	4	5	6	7	8	9	10	11	12
Sex	F	M	F	M	M	F	M	M	F	M	F	M
Duration of illness	15	14	25	30	20	22	10	5	20	10	18	5
Number of previous ineffective pharmacotherapy trials prior to the use of clozapine + cariprazine combination	5	6	12	14	7	11	5	11	6	6	4	8
Dose of clozapine that was combined with cariprazine	300 mg	200 mg	300 mg	500 mg	375 mg	150 mg	400 mg	200 mg	425 mg	400 mg	100 mg	200 mg
Antipsychotic used in combination with clozapine prior to switch to lurasidone	Aripiprazole 30 mg	Risperidone long-acting injection (25 mg every 2 weeks)		Haloperidol and aripiprazole	Quetiapine 800 mg and cariprazine 4.5 mg	Cariprazine 3 mg and olanzapine 20 mg	Olanzapine 20 mg and amisulpride 800 mg	Amisulpride 200 mg	Haloperidol 8 mg	Ziprasidone 80 mg	Risperidone	Lurasidone, aripiprazole, and risperidone
Somatic comorbidities	Obesity, type 1 diabetes, hypothyroidism, and hypertension	—	Obesity and diabetes	Obesity and diabetes	Obesity, diabetes dyslipidemia, hyperuricemia, and supra-ventricular arrhythmia	—	Obesity, diabetes, and liver steatosis	—	—	Osteoporosis and hyperparathyroidism	Obesity	—
Addictions (ICD-10 code)	No	No	No	No	No	No	No	No	No	No	No	No
Other psychotropic medications used at the time of adding the antipsychotic (reason for their use)	Sertraline 50 mg	Trazodone 150 mg	Pregabalin 300 mg	Biperiden and sertraline 100 mg	Clonazepam 3 mg, lamotrigine 100 mg, and pregabalin 450 mg	Lorazepam 5 mg and trazodone 100 mg	Alprazolam 3 mg	Lamotrigine 200 mg, pregabalin 200 mg, and biperiden 4 mg	Pregabalin 300 mg, biperiden 4 mg, and lorazepam 2 mg	Estazolam 2 mg	—	Pregabalin 150 mg, lamotrigine 200 mg, and alprazolam 5 mg
Non-psychiatric medications (with daily doses)	Insulin, metoprolol, metformin, and olmesartan	—	Metformin	Metformin	Dabigatran 300 mg, metoprolol 100 mg, amlodipine 10 mg, metformin 2,000 mg, allopurinol 300 mg, and rosuvastatin 20 mg	Bisoprolol 2.5 mg	Bisoprolol 5 mg, ursodeoxycholic acid 1,000 mg, and ramipril 2.5 mg	—	Bisoprolol 5 mg	Bisoprolol 5 mg, torsemide 5 mg, and vitamin D 1000 iu	—	—
Initial dose of cariprazine	1.5 mg	3 mg	3 mg	3 mg	1.5 mg	1.5 mg	1.5 mg	1.5 mg	1.5 mg	1.5 mg	1.5 mg	1.5 mg
Final dose of cariprazine	4.5 mg	4.5 mg	4.5 mg	4.5 mg	4.5 mg	6 mg	6 mg	4.5 mg	4.5 mg	3 mg	3 mg	4.5 mg
Duration of the combined treatment (clozapine + cariprazine), in months	9	10	8	4	4	3/4	2	10	3/4	8	12	15
Number of weeks until the observable therapeutic effect was achieved	4 (non-significant improvement) and 12 (significant improvement)	8 (non-significant improvement) and 16 (very significant improvement)	6	5	4	-	-	16	-	16	4	12
Effects of the addition of cariprazine (descriptive)	Reduction in negative, positive, and mood symptoms	Reduction in negative and anxiety symptoms. Improvement in global functioning in terms of overall activity, independence, ability to make purchases independently, participation in household activities, and overall decrease in the time spent in bed	Reduction in negative and affective symptoms	Reduction in anxiety symptoms and improvement in global functioning in terms of overall activity	Improvement in formal thought disorders, better social contacts and communication, higher activity, less severe positive symptoms (hallucinations and delusions), and better self-care	Exacerbation of akathisia	Increase in anxiety and agitation	Improvement in global functioning, decreased positive and negative symptoms, less anxiety, and improved cognitive functions	Increased anxiety and intensification of delusions	Reduction in positive symptoms	Reduction in positive and negative symptoms that enabled reduction of the clozapine daily dose, leading to a decrease in the severity of side effects (somnolence and sedation)	Reduction in negative and residual positive symptoms

**TABLE 3:** Summarized description of the schizophrenia patient group treated with cariprazine augmentation of clozapine.

	Treatment-resistant schizophrenia patients undergoing cariprazine augmentation of clozapine treatment (n = 12)
Age [median number of years, (25th percentile–75th percentile)]	38 (27.5–45)
Sex (women/men)	5/7
Duration of illness [median number of years, (25th percentile–75th percentile)]	16.5 (10–21.5)
Number of previous unsuccessful pharmacotherapies before introducing lurasidone augmentation of clozapine [median number of trials (25th percentile–75th percentile)]	6 (5–9.5)
Initial cariprazine dose [mean mg, (SD)]	1.88 (0.5)
Final cariprazine dose [mean mg, (SD)]	4.5 (1.06)
Clozapine dose [mean mg, (SD)]	295.84 (142.95)
Duration of cariprazine augmentation of clozapine treatment [median number of weeks, (25th percentile–75th percentile)]	32 (10–40)
Number of weeks of treatment until observable therapeutic effects [median, (25th percentile–75th percentile)]	6 (4–12)
Number of patients responding to treatment [n, (%)]	9 (75%)
Discontinuation of treatment due to side effects [n, (%)]	1 (8.3%)
Discontinuation due to symptom exacerbation [n, (%)]	2 (16.67%)

**TABLE 4:** Characteristics of the psychopathological symptoms and somatic conditions presented by schizophrenia patients before initiation of cariprazine augmentation of clozapine.

Case number	1	2	3	4	5	6	7	8	9	10	11	12
Residual positive symptoms	x	x		xx	x	x		x	x		x	x
Exacerbation of positive symptoms							x			x		
Negative symptoms	xxx	xxx	xxx	xxx	x	x	x	x	x		x	x
Depressive symptoms	x		xx			x		x				
Anxiety		x		x		x	x	x	x	x		
Suicidal thoughts												
Cognitive dysfunctions		x		xx	x	x		x				
Sexual dysfunctions								x				
Hyperprolactinemia										x		
Increased appetite and body weight/obesity	x		xx	xx	x		x					
Disorders of glucose metabolism	x		x	x	x		x					

xxx, high severity; xx, moderate severity; x, low severity.

In nine out of twelve patients, cariprazine replaced other antipsychotic drugs that were used in combination with clozapine, particularly aripiprazole (one patient), risperidone (two patients: one treated with the use of oral tablets and the other with the use of long-acting injections), haloperidol (two patients), amisulpride (two patients), ziprasidone (one patient), and lurasidone (one patient). In one case, cariprazine was added to clozapine monotherapy. In two patients, clozapine replaced the other antipsychotic drugs that were used in combination with cariprazine, particularly olanzapine (one patient) and quetiapine (one patient).

The mean daily dose of clozapine that was used with cariprazine was 295.83 ± 142.95 mg (dose range 100–500 mg). In every case, the clozapine serum concentration remained at a therapeutic level. The mean initial and final doses of cariprazine added to clozapine were 1.88 ± 0.5 mg (dose range 1.5–3 mg) and 4.5 ± 1.06 mg (dose range 3–6 mg), respectively. The duration of cariprazine augmentation of clozapine ranged from 0.5 to 15 months (median: 8 months).

A positive therapeutic effect (CGI-I = 2) was observed in nine out of twelve patients (75%) after 4–12 weeks of treatment (median 6). Medical records revealed that the patients showed an observable improvement in terms of negative symptoms (seven patients), positive symptoms (five patients), general functioning (six patients), affective symptoms (two patients), anxiety (two patients), and cognitive functions (one patient). In one patient, a decrease in the severity of positive and negative symptoms allowed the clozapine dose to be reduced, which contributed to the reduction of sedation and somnolence. In three patients, exacerbation of symptoms was observed during the treatment using a combination of cariprazine and clozapine, particularly anxiety (two patients), agitation (one patient), delusions (one patient), and akathisia (one patient).

## 4 Discussion

Our study presents the first retrospective chart review of cariprazine augmentation of clozapine effects in the management of treatment-resistant schizophrenia. We demonstrated a thorough clinical description of 12 schizophrenia patients treated using a combination of clozapine and cariprazine for a period ranging from 3 weeks to 15 months. Despite the high level of treatment resistance, the majority of the patients (9/12, 75%) presented a therapeutic response, resulting in an improvement in terms of negative, positive, affective, and anxiety symptoms, as well as global functioning.

Taking into account that treatment-resistant schizophrenia is associated with severe negative symptoms, and the fact that cariprazine is particularly effective in their improvement, it is surprising that so few studies assessed the efficacy of the cariprazine augmentation of clozapine in this clinical group (Iasevoli et al., 2018; Misiak et al., 2018). Pappa et al. (2022) evaluated the use of this combination in a heterogeneous group of 10 patients with a non-satisfactory response to clozapine, which consisted of 6 patients with schizophrenia, 3 with schizoaffective disorder, and 1 with personality disorder with autism spectrum disorder. Cariprazine was introduced due to inadequate treatment response, persistent negative symptoms, clozapine-induced side effects, and/or lack of tolerance to previous augmentation strategies (Pappa et al., 2022). During the 3 months of the treatment, the authors observed a significant reduction in terms of positive, negative, and total Positive and Negative Syndrome Scale (PANSS) scores (Pappa et al., 2022). Similarly, case studies indicated that the combination of cariprazine and clozapine may lead to a marked reduction in negative and positive symptoms and improvement of patient global functioning (De Berardis et al., 2021; Bogren et al., 2022; Oloyede et al., 2022).

Our study presents the largest up-to-date sample of treatment-resistant schizophrenia patients treated with the cariprazine augmentation of clozapine. While most of the previous reports focused on the beneficial effect of this combination on negative symptoms, we showed that cariprazine augmentation of clozapine may also lead to a significant improvement across positive, depressive, and anxiety symptoms, as well as in global and cognitive functioning, despite a high number of previously failed treatment trials. Those results are in line with the observations from the four cases, indicating that cariprazine was effective where other augmentation antipsychotics failed, including the often preferred amisulpride (Oloyede et al., 2022). Case reports indicate that the introduction of cariprazine may reduce the severity of negative and positive symptoms to such an extent that it allows clozapine reduction (De Berardis et al., 2021) or even discontinuation (Aubel, 2021). In our study, we identified a similar case of a patient in whom the introduction of cariprazine led to a significant reduction in those symptoms and enabled the clozapine dose to be decreased. This contributed to the reduction of sedation and somnolence associated with the use of this drug.

We showed that cariprazine augmentation of clozapine was relatively well tolerated. Three out of twelve patients (25%) discontinued the treatment due to the exacerbation of symptoms or the occurrence of side effects. In one patient, there was an observable increase in anxiety and agitation. A second patient revealed the intensification of delusions with concomitant anxiety. Side effects (akathisia) occurred in only one patient. The management of the adverse events relied on the discontinuation of the cariprazine treatment, which resulted in amelioration of those symptoms in every mentioned case. Tolerance rates in our retrospective chart review were comparable to those presented in the much shorter study by Pappa et al. (2022), in which 80% of the patients adhered to the treatment throughout the 3 months of the treatment. In the aforementioned study, one patient developed dizziness that required dose optimization, while two patients discontinued the treatment due to poor response or restlessness. However, overall cariprazine augmentation was well tolerated by the rest of the group, including patients in whom previous treatment strategies had to be changed due to the lack of tolerance of side effects (Pappa et al., 2022).

Our study significantly contributes to the evidence supporting the use of modern antipsychotics as augmentation agents of clozapine in treatment-resistant schizophrenia (Siwek et al., 2023). While the observation time in previous studies was limited to 3 months (Pappa et al., 2022), our retrospective chart review presents the cases in which the duration of the combined treatment reached up to 15 months (median 8 months). Long-term observations are crucial in terms of evaluating cariprazine efficacy, especially in the area of negative symptoms. For instance, a 26-week-long, double-blind, placebo-controlled randomized trial comparing the effects of this drug with those of risperidone has shown that statistically significant difference in favor of cariprazine appears only after 14 weeks of treatment (Németh et al., 2017). In our study, the therapeutic effect of cariprazine augmentation of clozapine was observable after a median of 8 weeks. It is noteworthy that in four patients, significant improvement emerged after 4 months of therapy. Thus, it is crucial for future studies evaluating the efficacy of cariprazine augmentation of clozapine to implement long-term observations of patient treatment.

Positive effects of cariprazine augmentation of clozapine treatment may be associated with the significant pharmacodynamic complementarity of both drugs. Both antipsychotics act as 5-HT_2a_ and 5-HT_7_ antagonists, but clozapine affinity to those receptors is significantly higher. Unlike clozapine, cariprazine is a D_2_/D_3_ partial agonist with unique high affinity to D_3_ receptors. This feature allows this drug to elicit differential activities depending on the synaptic environment. Particularly, it inhibits the receptor in the presence of agonists with higher intrinsic activity but acts as an agonist in their absence (Misiak et al., 2018). Cariprazine activity related to D_3_ receptors is associated with the improvement in positive, negative, and depressive symptoms, as well as with procognitive effects (Misiak et al., 2018). Contrary to clozapine, cariprazine presents negligible affinity to M_1_, α_1_, and α_2_ receptors and low affinity to H_1_ receptors, which is associated with the low risk of sedation, metabolic side effects, and hypotension (Misiak et al., 2018). Co-administration of those antipsychotics is associated with the low risk of significant pharmacokinetic interactions. Cariprazine metabolites are eliminated mainly by CYP3A4, and they act as weak competitive inhibitors of CYP2D6 and CYP3A4; thus, they have a minimal impact on clozapine, which is extensively metabolized via CYP1A2 (Mauri et al., 2018; Misiak et al., 2018). We are aware of the limitations of our study: the heterogeneous group of the analyzed patients (e.g., variability in the duration of treatment and differences in terms of the history of the unsuccessful treatment trials), a relatively small number of cases, no data on socioeconomic background, lack of a control group, and no access to the clozapine blood levels of most of the patients. Our study did not implement the use of specific clinical tools measuring negative, positive, anxiety, or affective symptoms or measures of cognitive and global functioning. Data regarding the abovementioned areas were derived from patient medical documentation. Being a retrospective chart review, the study might have inherent biases, such as selection bias and information bias. Those limitations decrease the strength of the conclusions that can be drawn from our study. However, we believe that due to the significant research gap in the area of a clozapine augmentation strategy with the use of modern antipsychotics, our studies provide valuable data that can be used in future randomized controlled trials addressing this issue.

To the best of our knowledge, our study presents a thorough description of the largest number of treatment-resistant schizophrenia patients medicated using cariprazine augmentation of clozapine in a “real-world” setting. The majority of the analyzed cases responded to the combination of the abovementioned drugs with a relatively good tolerance to the treatment. The use of cariprazine augmentation of clozapine was associated with an observable improvement in patient global functioning as well as a decrease in the severity of positive, negative, depressive, and anxiety symptoms. It is noteworthy that this effect was observed in the cases of individuals who underwent more than 10 ineffective antipsychotic treatment trials. Our preliminary study points out the need of well-designed, long-term, randomized clinical trials evaluating the efficacy of the cariprazine augmentation of clozapine in a group of treatment-resistant schizophrenia patients.

## Data Availability

The original contributions presented in the study are included in the article/Supplementary Material, further inquiries can be directed to the corresponding authors.
