# Supplementary material for: Cariprazine augmentation of clozapine in schizophrenia—a retrospective chart review
Source: Front Pharmacol. 2024 Jan 4;14:1321112. doi: 10.3389/fphar.2023.1321112 (PMC10794393; doi:10.3389/fphar.2023.1321112)
Supplement: Supplementary file 1 [file Table1.DOCX]

Annex 1

Data collecting form for all patients on clozapine:

| Patient code | Age | Gender | Diagnosis | Clozapine dose (mg) | Treatment schedule (1 monotherapy; 2-politherapy) | Other antipsychotics with dosing |
| --- | --- | --- | --- | --- | --- | --- |
|  |  |  |  |  |  |  |

Form for patients on clozapine and cariprazine combination:

| Case number |  |
| --- | --- |
| Illness duration (in years) |  |
| Number of previous ineffective pharmacotherapy trials prior to the use of clozapine + cariprazine combination |  |
| Antipsychotic used in combination with clozapine prior to switch to cariprazine |  |
| Somatic comorbidities |  |
| Addictions |  |
| Other psychotropic medications used at the time of adding the antipsychotic |  |
| Nonpsychiatric medications (with daily doses) |  |
| Initial dose of cariprazine |  |
| Target dose of cariprazine |  |
| Duration of the combined treatment (clozapine + cariprazine), in months |  |
| Clozapine dose (mg) |  |
| **Symptoms at the beginning of the combined clozapine + cariprazine treatment:** | |
| Residual positive symptoms (1-yes; 0-no) |  |
| Exacerbation of positive symptoms (1-yes; 0-no) |  |
| Negative symptoms (1-yes; 0-no) |  |
| Depressive symptoms (1-yes; 0-no) |  |
| Anxiety (1-yes; 0-no) |  |
| Suicidal thoughts (1-yes; 0-no) |  |
| Cognitive dysfunctions (1-yes; 0-no) |  |
| Sexual dysfunctions (1-yes; 0-no) |  |
| Hyperprolactinemia (1-yes; 0-no) |  |
| Overweight/Obesity (1-yes; 0-no) |  |
| Disorders of glucose metabolism (1-yes; 0-no) |  |
